# Effects of plasma expansion with albumin and paracentesis on haemodynamics and kidney function in critically ill cirrhotic patients with tense ascites and hepatorenal syndrome: a prospective uncontrolled trial

**DOI:** 10.1186/cc6765

**Published:** 2008-01-15

**Authors:** Andreas Umgelter, Wolfgang Reindl, Katrin S Wagner, Michael Franzen, Konrad Stock, Roland M Schmid, Wolfgang Huber

**Affiliations:** 1 Medizinische Klinik und Poliklinik der Technischen Universität München, Ismaningerstrasse 22, 81675 München, Germany; 2 Klinik für Kardiologie und Internistische Intensivmedizin, Klinikum Bogenhausen, Städtisches Klinikum München GmbH, Englschalkinger Strasse 77, 81925 München, Germany

## Abstract

**Introduction:**

Circulatory dysfunction in cirrhotic patients may cause a specific kind of functional renal failure termed hepato-renal syndrome (HRS). It contributes to the high incidence of renal failure in cirrhotic intensive care unit (ICU) patients. Fluid therapy may aggravate renal failure by increasing ascites and intra-abdominal pressure (IAP). This study investigates the short-term effects of paracentesis on haemodynamics and kidney function in volume resuscitated patients with HRS.

**Methods:**

Nineteen consecutive cirrhotic patients with HRS were studied. Circulatory parameters and renal function were analysed before and after plasma expansion and paracentesis. Haemodynamic monitoring was performed by transpulmonary thermodilution.

**Results:**

After infusion of 200 ml of 20% human albumin solution, mean arterial pressure (MAP) and central venous pressure remained unchanged. Global end-diastolic volume index (GEDVI) increased from 791 ml m^-2 ^(693 to 862) (median and 25th to 75th percentile) to 844 ml m^-2 ^(751 to 933). Cardiac index (CI) increased from 4.1 l min^-1 ^m^-2 ^(3.6 to 5.0) to 4.7 l min^-1 ^m^-2 ^(4.0 to 5.8), whereas systemic vascular resistance index (SVRI) decreased from 1,422 dyn s cm^-5 ^m^-2 ^(1,081 to 1,772) to 1,171 dyn s cm^-5 ^m^-2 ^(893 to 1,705). Creatinine clearance (CC) and fractional excretion of sodium (FeNa) were not affected. During paracentesis, IAP decreased from 22 mmHg (18 to 24) to 9 mmHg (8 to 12). MAP decreased from 81 mmHg (74 to 100) to 80 mmHg (71 to 89), and CI increased from 4.1 l min^-1 ^m^-2 ^(3.2 to 4.3) to 4.2 l min^-1 ^m^-2 ^(3.6 to 4.7), whereas SVRI decreased from 1,639 dyn s cm^-5 ^m^-2 ^(1,168 to 2,037) to 1,301 dyn s cm^-5 ^m^-2 ^(1,124 to 1,751). CC during the 12-hour interval after paracentesis was significantly higher than during the 12 hours before (33 ml min^-1 ^(16 to 50) compared with 23 ml min^-1 ^(12 to 49)). CC remained elevated for the rest of the observation period. FeNa increased after paracentesis but returned to baseline levels after 24 hours.

**Conclusion:**

Paracentesis with parameter-guided fluid substitution and maintenance of central blood volume may improve renal function and is safe in the treatment of ICU patients with hepato-renal failure.

## Introduction

According to the hypothesis of arterial vasodilation, portal hypertension in cirrhotic patients leads to arterial vasodilation in extra-renal vascular beds, especially in the splanchnic system, and to the abdominal pooling of blood [1,2]. These result in a decreased effective blood volume in the central circulation and relative hypovolaemia. This haemodynamic dysfunction is common in patients with cirrhosis and gives rise to the compensatory stimulation of endogenous vasopressor systems such as the renin–angiotensin–aldosterone system, the vasopressin system and the sympathetic nervous system. These become increasingly strained with a narrowing capacity to cope with additional insults such as haemorrhage, infection or overzealous use of diuretics. Activation of systemic vasopressor systems causes renal vasoconstriction that puts those patients at risk of acute pre-renal kidney failure, which contributes substantially to the mortality risk in critically ill cirrhotic patients [3]. Cirrhotic intensive care unit (ICU) patients with acute renal failure (ARF) may be classified into three groups: patients with structural kidney disease such as glomerulonephritis, vasculitis or acute tubular necrosis, patients with non-specific causes of pre-renal failure, and patients with functional renal failure specific to the circulatory dysfunction of cirrhotic patients, termed hepato-renal syndrome (HRS) [4].

Whereas the role of fluid resuscitation has been extensively investigated in non-cirrhotic patients with sepsis-associated circulatory failure, data on fluid resuscitation in cirrhotic patients with this specific type of pre-renal kidney failure are scarce. One problem with fluid expansion in cirrhotic patients lies in the loss of infused volume to the intra-peritoneal space, where it increases intra-abdominal pressure (IAP). The presence of ascites itself is closely related to the development of renal failure, and 20% of cirrhotic patients with tense ascites develop HRS. Intra-abdominal pressure may impair renal perfusion by decreasing the renal perfusion pressure (RPP) and filtration gradient (FG) [5]. In addition an increase in IAP could decrease venous return to the right ventricle or impair right-ventricular diastolic filling, thus aggravating the hyperdynamic circulatory dysfunction by adding a hypovolaemic or obstructive component.

Several studies focused on the prevention of post-paracentesis circulatory dysfunction [6-8] or on the prevention of hepato-renal failure in patients with spontaneous bacterial peritonitis [9]. For both indications, plasma expansion with human albumin has become firmly established. The treatment of HRS, once it has occurred, has been addressed by other studies, mainly focusing on the effect of vasopressors [10,11], but suggesting that plasma expansion with albumin may be an important part of the treatment [12].

The present study was undertaken in cirrhotic intensive care patients with advanced cirrhosis, tense ascites and acute renal failure that persisted after fluid resuscitation but without evidence of intrinsic kidney disease. The aim was to investigate the single and combined haemodynamic and renal effects of plasma expansion by infusion of albumin and of the decrease in intra-abdominal pressure by paracentesis under the condition of parameter-guided maintenance of central volume.

## Methods

### Definitions

The – recently amended – definition of HRS has known setbacks and is especially difficult to apply in ICU patients. The two groups, HRS 1 and HRS 2, are delineated by criteria that are not congruent: whereas HRS 1 is defined by an acute increase in serum creatinine to a level above 221 μmol l^-1 ^and HRS 2 by a slow increase in creatinine to above 133 μmol l^-1^, the classification of patients with an acute renal failure who do not reach a serum creatinine over 221 μmol l^-1 ^is difficult to do adequately. Likewise, the delimitation from septic kidney failure is fuzzy. For the purpose of this study, HRS was defined as kidney failure in cirrhotic patients who had documented normal serum creatinine values before ICU admission and who had suffered an acute increase in serum creatinine to values above 133 μmol l^-1 ^within less than 14 days that persisted despite resolution of the precipitating event and despite adequate haemodynamic management and who showed no evidence of intrinsic kidney disease or current infection.

### Patients

Patients were included if they had persistent acute kidney failure (serum creatinine > 133 μmol l^-1^) with a previously normal kidney function (serum creatinine < 98 μmol l^-1^), caused by an acute condition treated in our intensive care department and if they had a stable serum creatinine (less than 10% change per 24 hours) in the 24 hours preceding the study period. Patients had to be haemodynamically stable without vasopressors or positively inotropic substances for 2 days after treatment of the condition leading to ICU admission without an improvement in kidney function, and they had to fulfil the diagnostic criteria established by the International Ascites Club in 1994 (Table 1) [13]. Current infection was excluded by obtaining microbiological cultures of blood and urine and by ascitic cell differentiation. Thrombosis of the portal vein was excluded in each patient by duplex ultrasound. Patients were also excluded if there was any evidence of primary kidney disease found by screening ultrasound or biochemical and biochemical and microscopic analysis of urine or if the fractional excretion of sodium (FeNa) was more than 1%, indicating other than haemodynamic causes. None of the patients had received diuretics, aminoglycosides or vancomycin for at least 1 week before inclusion, and all had received adequate volume resuscitation during the treatment of their precipitating condition.

**Table 1:** International Ascites Club's definition of hepato-renal syndrome

Chronic or acute liver disease with advanced hepatic failure and portal hypertension
Low glomerular filtration rate, as indicated by serum creatinine of more than 1.5 mg dl^-1 ^or 24-hour creatinine clearance less than 40 ml min^-1^
Absence of shock, ongoing bacterial infection, and current or recent treatment with nephrotoxic drugs. Absence of gastrointestinal fluid losses (repeated vomiting or intense diarrhoea) or renal fluid losses (weight loss more than 500 g per day for several days in patients with ascites without peripheral oedema or 1,000 g per day in patients with peripheral oedema)
No sustained improvement in renal function (decrease in serum creatinine to 1.5 mg dl^-1 ^or less, or increase in creatinine clearance to 40 ml min^-1 ^or more) after diuretic withdrawal and expansion of plasma volume with 1.5 litres of isotonic saline
Proteinuria less than 500 mg dl^-1 ^and no ultrasonographic evidence of obstructive uropathy or parenchymal renal disease

Catheters for invasive haemodynamic monitoring had to be in place, and written consent was obtained from the patients or their next of kin. Our institutional ethics committee approved the study.

### Haemodynamic measurements and measurements of intra-abdominal pressure

Patients were studied in a supine position, with zero pressure at the mid-axillary line. Haemodynamic monitoring by transpulmonary thermodilution was begun during the initial critical care treatment. We used a commercially available system (PiCCO; PULSION Medical Systems, Munich, Germany), which works by the injection of a cold bolus of normal saline through a central venous line that is detected after passing through the cardiac chambers, pulmonary vasculature and part of the aorta by a thermistor-tipped arterial line inserted into one of the femoral arteries and advanced to the aortic bifurcation. The mean transit time and the slope of the temperature curve at the thermistor permit the assessment of cardiac output as well as that of the amount of intra-thoracic volume that has been passed through and also the pulmonary blood volume. Subtracting pulmonary blood volume from intra-thoracic blood volume provides an estimation of the largest volume of blood contained in the four heart chambers, called, after indexing by body surface area, the global end-diastolic volume index (GEDVI). All measurements were made in triplicate and averaged. They were performed at 12-hour intervals and immediately before and after the infusion of a fluid load and before and after paracentesis.

Intra-abdominal pressure was measured at the beginning and end of paracentesis by connecting the drainage tube to a pressure transducer with the zero level set at the mid-axillary line. Measurements were recorded after some equilibration time and after verification of ventilatory modulation of the readings, at end-expiration. RPP and renal FG were determined from RPP = MAP - IAP and FG = MAP - (2 × IAP) [14], where MAP is mean arterial pressure.

### Assessment of kidney function

Urinary output was recorded and urine was collected over 12-hour intervals corresponding to those of haemodynamic measurements, and blood was taken at the end of each 12-hour interval. After biochemical analysis, FeNa and creatinine clearance (CC) were calculated from standard formulae.

### Study protocol

**Figure 1:**
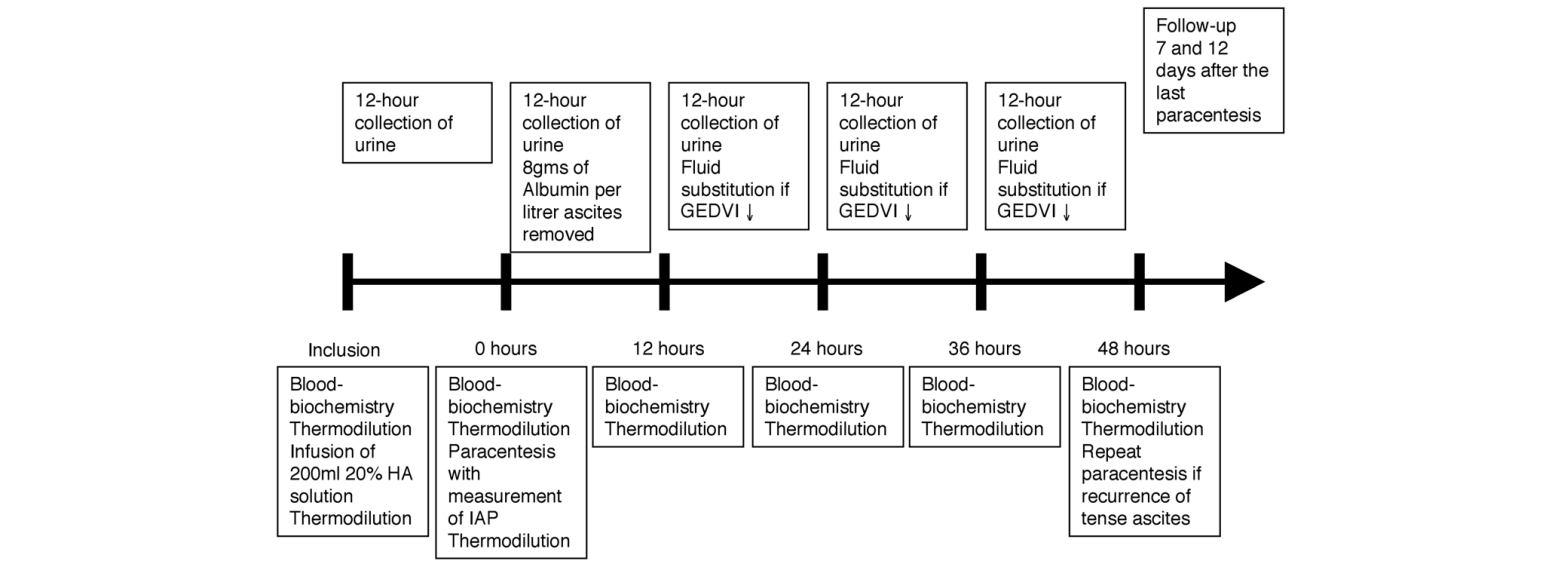
Flow-chart of the protocol. GEDVI, global end-diastolic volume index; HA, human albumin; IAP, intra-abdominal pressure.

Immediately after inclusion, patients received an infusion of 200 ml of 20% human albumin solution. Haemodynamic measurements by transpulmonary thermodilution were performed before and after infusion and after 12 hours. After this first 12-hour interval, paracentesis was performed with measurements of intra-abdominal pressure and haemodynamic parameters before and after paracentesis. Thereafter, patients received albumin solution up to a total of 8 g of albumin per litre of ascites removed, and saline thereafter. Fluid therapy was titrated so as to keep GEDVI and cardiac index (CI) constant. Urine was collected over four 12-hour intervals for the determination of CC and FeNa. Thereafter, paracentesis could be repeated if clinically indicated (for example by dyspnoea or pain) and if there had been no increase in serum creatinine after the first intervention. Monitoring and measurements were performed as before. Follow-up measurements of serum creatinine were made 7 and 12 days after the last paracentesis (Figure 1).

### Statistical tests

We used the Kolmogorov–Smirnov test to examine the distribution of data. Because it emerged that data for most parameters were not normally distributed, the Wilcoxon test was used for comparisons of paired data. To avoid false positive results resulting from multiple testing, the level of significance was adjusted according to Bonferroni when data from multiple time points were compared with baseline values. *P *< 0.05 was regarded as indicating significance. SPSS 11 for MAC was used for the calculations. Correlations between haemodynamic and renal parameters were analysed with Spearman's non-parametric test.

## Results

Nineteen consecutive patients (17 male, 2 female; age 59 ± 8.6 years (mean ± SD) were included between September 2004 and August 2005. 14 of these were listed for liver transplantation. Cirrhosis was due to alcohol (*n *= 14), chronic hepatitis C (*n *= 2), chronic hepatitis B (*n *= 1) or cryptogenic (*n *= 2). The acute conditions leading to ICU admission were spontaneous bacterial peritonitis (*n *= 7), sepsis of other origin (*n *= 6) and variceal haemorrhage (*n *= 5). One patient was admitted because of hepatic encephalopathy, hypotension and acute kidney failure. Patients' baseline parameters are presented in Table 2.

**Table 2:** Patients' baseline parameters

Parameter	Value
Age (years)	60 (52–63)
Child–Pugh score	13 (10–14)
Serum creatinine (μmol l^-1^)	301 (168–451)
INR	1.5 (1.4–2.3)
Bilirubin (μmol l^-1^)	92 (26–329)
MELD score	23 (15–34)
Serum sodium (mmol l^-1^)	130 (126–136)
Serum albumin (g dl^-1^)	27 (18–29)
ASAT (U l^-1^)	103 (68–183)
ALAT (U l^-1^)	63 (35–91)
Haemoglobin (g dl^-1^)	9.4 (7.6–10.2)
MAP (mmHg)	79 (70–96)
CVP (mmHg)	9 (6–12)
GEDVI (ml m^-2^)	760 (717–906)
SVRI (dyn s cm^-5 ^m^-2^)	1,394 (1,161–2,037)
Cardiac index (l min^-1 ^m^-2^)	4.1 (3.2–4.4)

Data are presented as median (25th to 75th centile). INR, International Normalized Ratio; MELD, Model of End-Stage Liver Disease; ASAT, aspartate aminotransferase; ALAT, alanine aminotransferase; MAP, mean arterial pressure; CVP, central venous pressure; GEDVI, global end-diastolic volume index; SVRI, systemic vascular resistance index.

### Immediate haemodynamic and renal effects of plasma expansion

The haemodynamic changes after a fluid load of 200 ml of 20% human albumin solution are presented in Table 3. Central blood volume increased, and there was a significant decrease in peripheral vascular resistance. MAP remained unchanged and, consequently, there was a rise in CI. During the following 12-hour period there was no change in CC and FeNa.

**Table 3:** Immediate effects of plasma expansion with 200 ml 20% human albumin solution (*n *= 19)

Parameter	Before plasma expansion	After plasma expansion	*P*
MAP (mmHg)	82 (69–92)	83 (70–89)	0.852
CVP (mmHg)	9 (7–12)	10 (7–13)	0.205
GEDVI (ml m^-2^)	791 (693–862)	844 (751–933)	0.001
SVRI (dyn s cm^-5 ^m^-2^)	1,422 (1,081–1,772)	1,171 (893–1,705)	0.006
CI (l min^-1 ^m^-2^)	4.1 (3.6–5.0)	4.7 (4.0–5.8)	<0.001
CC (ml min^-1^) over 12 h	23 (16–38)	23 (16–50)	0.227
FeNa (%) over 12 h	0.04 (0.02–0.05)	0.04 (0.03–0.06)	0.152

Data are presented as median (25th to 75th centile). MAP, mean arterial pressure; CVP, central venous pressure; GEDVI, global end-diastolic volume index; SVRI, systemic vascular resistance index; CI, cardiac index; CC, creatinine clearance; FeNa, fractional excretion of sodium.

### Immediate haemodynamic and renal effects of paracentesis

**Figure 2:**
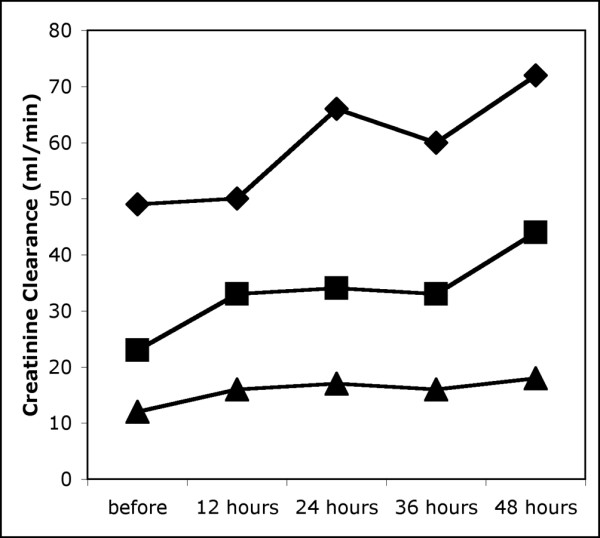
Creatinine clearance before and after paracentesis. The 25th, 50th and 75th centiles are given.

**Figure 3:**
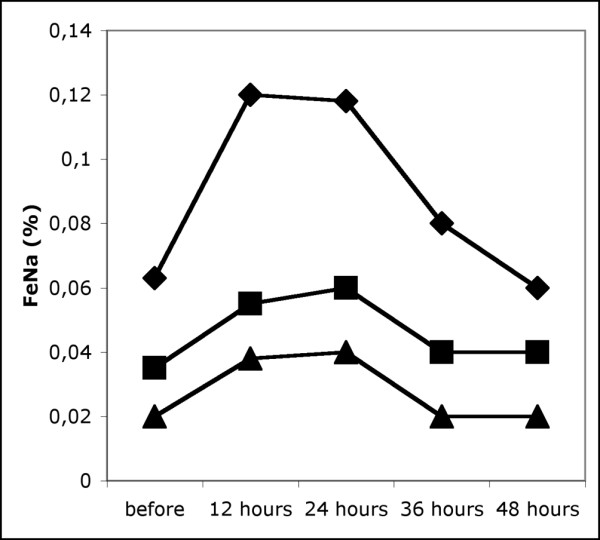
Fractional excretion of sodium before and after paracentesis. FeNa, fractional excretion of sodium. The 25th, 50th and 75th centiles are given.

Twenty-seven paracenteses were performed. One patient received five paracenteses, in four patients two paracenteses each were performed and one was performed in each of the remaining 14 patients. During the procedures, 6 litres (5.3 to 8.0) of ascites were removed. IAP fell from a median of 22 mmHg to a median of 9 mmHg (Table 4). Simultaneously, there was a significant, if small, decrease in MAP, central venous pressure (CVP) and systemic vascular resistance index (SVRI) and a small but consistent increase in CI. GEDVI remained unchanged. RPP increased significantly, and the associated increase in FG was substantial, amounting to 17 mmHg (7 to 21) (median and 25th to 75th percentile) or 34% (13 to 64). Simultaneously, there was a significant increase in CC and FeNa (Figures 2 and 3) during the following 12 hours. There were correlations between the initial level of IAP and the relative increase in CC during the 12 hours after paracentesis (*r *= -0.512, *P *= 0.018) as well as with the relative decrease in MAP immediately after paracentesis (*r *= -533, *P *= 0.013). The decrease in IAP was correlated with the relative change in SVRI after paracentesis (*r *= 0.586, *P *= 0.007).

**Table 4:** Immediate effects of large-volume paracentesis (*n *= 27)

Parameter	Before paracentesis	After paracentesis	*P*
IAP (mmHg)	22 (18–24)	9 (8–12)	<0.001
MAP (mmHg)	81 (74–100)	80 (71–89)	0.010
RPP (mmHg)	61 (53–79)	67 (60–81)	0.044
FG (mmHg)	42 (32–56)	55 (51–75)	0.001
CVP (mmHg)	11 (7–14)	9 (6–11)	0.014
GEDVI (ml m^-2^)	776 (717–917)	772 (702–875)	0.638
SVRI (dyn s cm^-5 ^m^-2^)	1,639 (1,168–2,037)	1,301 (1,124–1,751)	<0.001
CI (l min^-1 ^m^-2^)	4.1 (3.2–4.3)	4.2 (3.6–4.7)	0.001
CC (ml min^-1^)	23 (12–49)	33 (16–50)	0.002
FeNa (%)	0.035 (0.020–0.063)	0.055 (0.038–0.120)	0.001

Data are presented as median (25th to 75th centile). IAP, intra-abdominal pressure; MAP, mean arterial pressure; RPP, renal perfusion pressure; FG, filtration gradient; CVP, central venous pressure; GEDVI, global end-diastolic volume index; SVRI, systemic vascular resistance index; CI, cardiac index; CC, creatinine clearance; FeNa, fractional excretion of sodium.

Repeat paracenteses were performed 60 hours (48 to 72) after the first paracentesis. IAP on repeat paracentesis was not significantly lower than on the respective previous paracentesis (23 mmHg (21 to 26) versus 25 mmHg (21 to 30), *P *= 0.056, *n *= 9).

### Haemodynamic parameters at 12, 24 and 48 hours after paracentesis

Fluid substitution after paracentesis was guided by transpulmonary thermodilution, with GEDVI used as reference variable. Including the volume load before paracentesis, patients received a total of 8 g (6.8 to 8.7) of albumin per litre of ascites removed. In addition, after paracentesis there was an hourly net fluid balance of +89 ml (61 to 101), resulting in infusion over 48 hours of 64% of the volume removed at paracentesis. GEDVI was kept constant despite a decrease in CVP that, however, failed to reach the level of significance. CI and MAP remained unchanged (Table 5).

**Table 5:** Time course of haemodynamic parameters and parameters of kidney function

Parameter	Before paracentesis	After paracentesis			
		
		12 h	24 h	36 h	48 h
MAP (mmHg)	81 (74–100)	80 (69–92)	77 (68–93)	74 (64–92)^a^	84 (75–96)
CVP (mmHg)	11 (7–14)	7 (4–11)	9 (7–13)	7 (5–13)	9 (7–12)
GEDVI (ml m^-2^)	776 (717–917)	750 (683–900)	810 (693–952)	838 (650–946)	798 (668–882)
SVRI (dyn s cm^-5 ^m^-2^)	1,639 (1,168–2,037)	1,552 (1,105–1,809)	1,487 (1,205–1,808)	1,381 (1,044–2,023)	1,591 (1,160–2,088)
CI (l min^-1 ^m^-2^)	4.1 (3.2–4.3)	3.9 (3.3–4.5)	4.1 (3.5–4.7)	3.8 (3.4–4.5)	3.9 (3.5–4.4)
CC (ml min^-1^)	23 (12–49)	33 (16–50)^a^	34 (17–66)^a^	33 (16–60)^a^	44 (18–72)^a^
FeNa %	0.035 (0.020–0.063)	0.055 (0.038–0.120)^a^	0.060 (0.040–0.118)^a^	0.040 (0.020–0.080)	0.040 (0.020–0.060)

Data are presented as median (25th to 75th centile). MAP, mean arterial pressure; CVP, central venous pressure; GEDVI, global end-diastolic volume index; SVRI, systemic vascular resistance index; CI, cardiac index; CC, creatinine clearance; FeNa, fractional excretion of sodium. ^a^*P *< 0.05 compared with baseline. Adjustment for multiple testing according to Bonferroni.

### Clinical outcome

The period of haemodynamic monitoring lasted for between 60 and 252 hours, depending on the number of paracenteses. Overall, there was decrease of serum creatinine from 300 μmol l^-1 ^(167 to 450) to 176 μmol l^-1 ^(88 to 256) in all patients. Values deteriorated in only two patients, in both after the first paracentesis. Both received terlipressin but did not respond and had to be dialysed. One was allotted a liver transplant and his renal function recovered thereafter; the other developed pneumonia and died in septic shock.

On day 7 after the last paracentesis, of the remaining 10 patients with an initial serum creatinine value of at least 221 μmol l^-1^, 6 had a serum creatinine of less than 221 μmol l^-1^, and two had a serum creatinine value of less than 133 μmol l^-1^. Four of them had a decrease in serum creatinine levels of more than 50%. On day 12, seven had a serum creatinine value of less than 221 μmol l^-1^, and three had a serum creatinine value of less than 133 μmol l^-1^. In five patients, the decrease in serum creatinine levels was larger than 50%.

**Figure 4:**
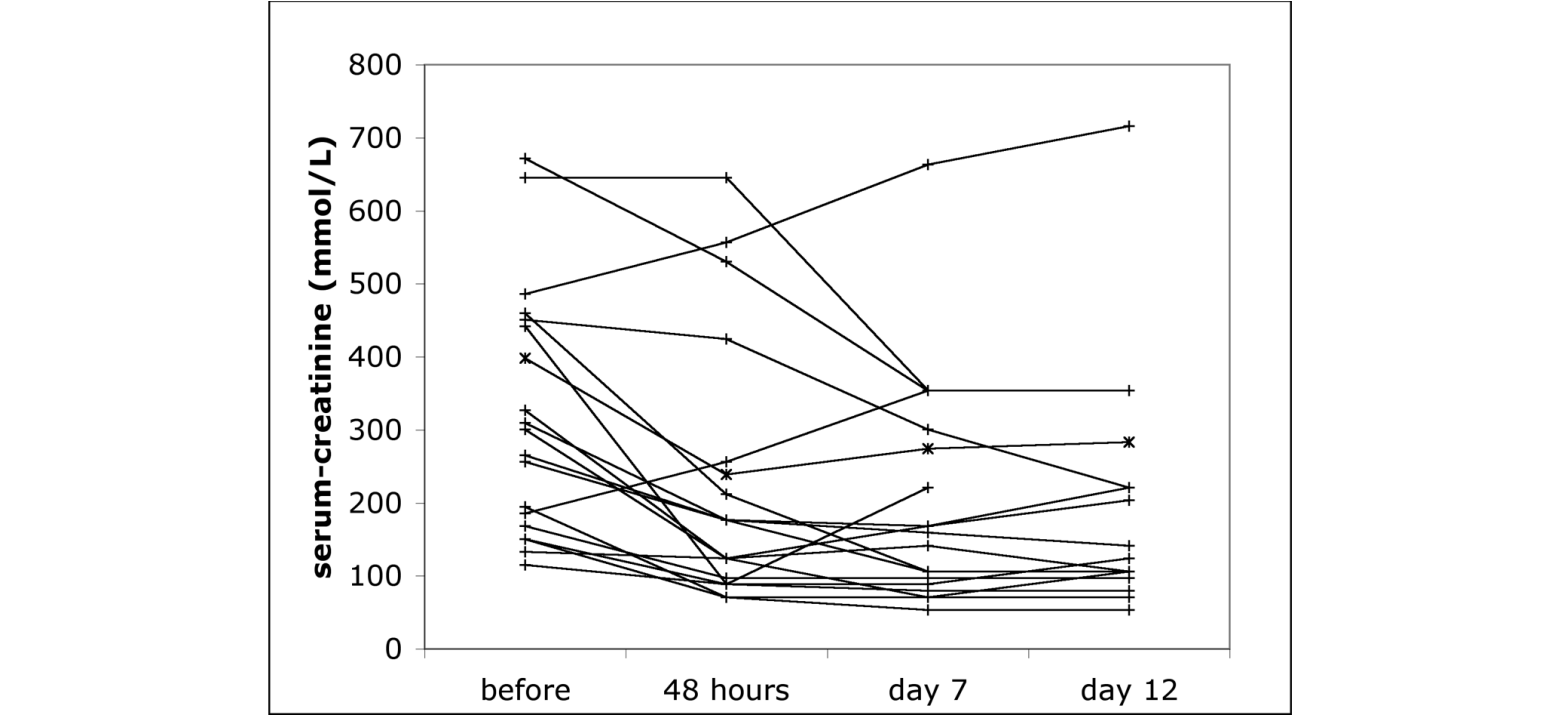
Serum creatinine levels before and after paracentesis. Absolute values for all patients before paracentesis and at 48 hours, 7 days and 12 days after the last paracentesis are presented.

Six of the seven patients with an initial serum creatinine level of between 133 and 221 μmol l^-1 ^had serum creatinine values of less than 133 μmol l^-1 ^on days 5 and 10 after the last paracentesis; in four, serum creatinine had normalized (Figure 4).

## Discussion

The results of the present study suggest that the decrease in intra-abdominal pressure achieved by paracentesis may be relevant for renal function in cirrhotic patients with renal failure and tense ascites. A limitation of this study is the lack of a control group. Therefore a causal relationship between paracentesis and the improvement in renal function cannot be proved. However, our demonstration that paracentesis did not result in a deterioration of kidney function caused by worsening circulatory dysfunction, in a cohort of patients receiving fluid therapy guided by transpulmonary thermodilution, may be relevant for the management of cirrhotic ICU patients. The application of the term HRS under the condition of ICU patients may be problematic, because contributing factors such as septic or post-ischaemic damage may not be strictly excluded by the diagnostic criteria applied. But in our opinion the term HRS is clinically useful to delineate a subset of cirrhotic patients with acute renal failure of a predominantly functional nature, that is in principle amenable to haemodynamic interventions. Accordingly, recent amendments to the definition have given up a strict delimitation of HRS from septic renal failure.

The patients in our study had persistent acute kidney failure despite adequate fluid resuscitation according to common criteria, as demonstrated by the fact that CVP and GEDVI were in the normal range. Nevertheless, after plasma expansion with 200 ml of a 20% human albumin solution, there was a further increase in GEDVI, indicating an increase in central blood volume, with the higher cardiac preload resulting in an increase in CI. Whereas MAP remained virtually unchanged, there was a substantial decrease in SVRI. This finding is in contrast with results of other studies investigating the effects of plasma expansion on haemodynamics in patients with cirrhosis. One actually reported an increase in SVRI after plasma expansion [15] and has been quoted as evidence for an indirect vasoconstrictor effect of albumin in cirrhotic patients in a current consensus statement on HRS [2]. In that study of patients with SBP, however, haemodynamic measurements were 5 days apart. In our opinion, the observed increase in SVRI was a consequence of resolution of the underlying septic vasodilation rather than an effect of the infused albumin. The authors of the second study described pooling of the infused volume in the mesenteric circulation in patients with advanced cirrhosis (Child–Turcotte class C) with no significant increase in central blood volume [16]. Still, the authors found an increase in CI with a concomitant decrease in SVRI. We believe that the trial may have been underpowered to detect a significant increase in central blood volume. In fact the data show an absolute increase of the same order of magnitude as that seen in our patients, but the former failed to reach the level of significance owing to the small number of patients (*n *= 9). The decrease in SVRI seen in our patients after plasma expansion may have been due to a decrease in activation of endogenous vasopressor systems. Several studies have described the circulatory dysfunction of cirrhotic patients as a primary peripheral arterial vasodilation and mesenteric blood pooling, resulting in a low effective arterial blood volume and compensatory stimulation of endogenous vasopressor systems. We did not measure the activity of vasopressor systems in our study, but decreased levels of renin and aldosterone have been reported in patients with Child–Pugh grade C cirrhosis after plasma expansion [16]. Despite the increase in CI, we did not see any change in kidney function after plasma expansion within the following 12-hour period. The observed haemodynamic changes may therefore have been too small to affect renal perfusion or may have been confined to other vascular beds. Results of a randomized study on the treatment of HRS comparing the efficacy of noradrenaline (norepinephrine) with that of terlipressin show that a substantial number of the patients initially screened responded to albumin alone if a CVP of between 10 and 15 cmH_2_O was obtained [12]. This compares to baseline values of 11 mmHg (14 cmH_2_O) in our patients, possibly indicating a larger central blood volume. However, CVP has been found to be unreliable as an indicator of preload and may be even less accurate in conditions with elevated intra-abdominal pressure [17-19]. The 15% increase in CI seen in our patients after a fluid load of 200 ml of human albumin solution shows that these patients were volume responsive despite their normal CVP. Larger doses of human albumin might thus have had more pronounced effects on renal perfusion.

Intra-abdominal hypertension was present in all patients, and paracentesis resulted in a substantial decrease in intra-abdominal pressure. Because the concomitant decrease in MAP was small, this resulted in a major increase in APP and FG. Simultaneously, CI increased while CVP and SVRI were reduced. This is in keeping with the results of previous studies assessing the effect of paracentesis on systemic haemodynamics [20-22]. These cardiocirculatory changes have been attributed to improved cardiac filling and an increased venous return after paracentesis. However, GEDVI, as a marker of preload, remained constant in our study, whereas CVP also decreased, arguing against a major contribution of increased cardiac preload to the rising CI. As MAP also decreased slightly, we believe it more likely that a decrease in afterload, as demonstrated by the falling SVRI, was the reason for the enhanced CI. This decrease in vascular resistance might have been the result of several factors. On the one hand, worsening circulatory dysfunction has been described after paracentesis [23]. On the other hand, release of IAP may increase splanchnic blood flow at lower pressures [22]. A reduction of IAP may also improve renal perfusion by lowering venous and retroperitoneal pressure. The observation, made by others, of decreasing serum renin levels after large-volume paracentesis supports the importance of this effect, because renin secretion is controlled by transmural arteriolar pressure [24] and hypoperfusion at the macula densa [25], both probably influenced by changes in intra-abdominal or retroperitoneal pressure. The increasing FeNa and CC seen in our patients adds further evidence to this concept and shows that the net result of immediate circulatory changes after paracentesis may be beneficial for renal function.

The elevation of CC seen already during the first 12 hours after paracentesis was maintained over 48 hours while central blood volume, as indicated by GEDVI, was kept constant. The improved serum creatinine values seen at 7 and 12 days after the last paracentesis also indicate that renal function remained above baseline in most of the patients. As has previously been shown, post-paracentesis circulatory dysfunction is most pronounced after 6 days [8]. Its detrimental effect on kidney function can be prevented by plasma expansion with albumin [8]. Our results suggest that elevated IAP may be a contributing factor in the development of renal failure in cirrhotic patients with tense ascites and that paracentesis may have a role in the treatment of HRS as long as central blood volume is maintained. The cause of the falling SVRI seen after paracentesis is controversial. On the basis of our findings we propose that increasing splanchnic and renal blood flow and decreased activation of endogenous vasopressor systems are important effects of paracentesis and that the decreased vascular tone may reflect not a deterioration of circulatory dysfunction but less demand for vasoconstrictor activation in the face of improving abdominal and renal perfusion pressures. The concomitant increase in splanchnic blood volume would further underline the importance of maintaining adequate preload, and post-paracentesis circulatory dysfunction could be regarded mainly as representing relative hypovolaemia caused by fluid losses into the intra-peritoneal compartment.

## Conclusion

This study indicates that the expansion of central blood volume is possible even in patients with advanced cirrhosis. The ensuing circulatory changes are small, however, and renal effects were not visible with the amount of albumin solution used in our study. After paracentesis there was a marked decrease in IAP and RPP. Under substitution of albumin and fluids to maintain central blood volume, there was a simultaneous improvement of renal function that may be relevant in patients with end-stage liver disease.

## Key messages

• Cardiac index in cirrhotic patients with hepato-renal syndrome may be fluid-responsive despite normal central venous pressure and global end-diastolic volume.

• Intra-abdominal hypertension, caused by ascites, can be reduced by paracentesis, resulting in a net increase in renal perfusion pressure.

• After paracentesis, fluid substitution can be titrated to keep global end-diastolic volume constant, and creatinine clearance and fractional excretion of sodium may increase.

• In cirrhotic intensive care patients with intra-abdominal hypertension caused by ascites resulting from fluid therapy, paracentesis is a safe procedure.

## Abbreviations

CI = cardiac index; CVP = central venous pressure; CC = creatinine clearance; FG = filtration gradient; FeNa = fractional excretion of sodium; GEDVI = global end-diastolic volume index; HRS = hepato-renal syndrome; IAP = intra-abdominal pressure; ICU = intensive care unit; MAP = mean arterial pressure; RPP = renal perfusion pressure; SVRI = systemic vascular resistance index.

## Competing interests

Andreas Umgelter and Wolfgang Huber have been speakers for Pulsion Medical Systems, Munich. The other authors declare no conflict of interest. There were no grants received for this study.

## Authors' contributions

The study was designed by AU and WH, who also co-wrote the manuscript. WR, KW, MF and KS were involved in patient management, acquisition and processing of data and revision of the manuscript. RMS was involved in designing the study and revised the manuscript. All authors read and approved the final manuscript.
